# Investigation of structural, electronic, mechanical, & optical characteristics of Ra based-cubic hydrides RbRaX_3_ (X= F and cl) perovskite materials for solar cell applications: First principle study

**DOI:** 10.1016/j.heliyon.2023.e18407

**Published:** 2023-07-17

**Authors:** Muhammad Khuram Shahzad, Muhammad Umair Farooq, Rashid Ali Laghari, Muhammad Aslam Khan, Muhammad Bilal Tahir, Waqar Azeem, Muhammad Mahmood Ali, Vineet Tirth

**Affiliations:** a Institute of Physics, Khwaja Fareed University of Engineering and Information Technology, Rahim Yar Khan, 64200, Pakistan; b Center of Theoretical and Computational Research, Khwaja Fareed University of Engineering and Information Technology, Rahim Yar Khan, Pakistan; c Institute of Physics, The Islamia University of Bahawalpur, Bahawalpur 63100, Pakistan; d Interdisciplinary Research Center for Intelligent Manufacturing and Robotics, King Fahd University of Petroleum and Minerals, Dhahran, 31261, Saudi Arabia; e Faculty of Resilience, Rabdan Academy, Abu Dhabi, United Arab Emirates; f Department of Mechatronic Engineering, Atlantic Technological University Sligo, Ash Lane, F91 YW50 Sligo, Ireland; g Centre for Mathematical Modeling and Intelligent Systems for Health and Environment (MISHE), Atlantic Technological University Sligo, Ash Lane, F91 YW50 Sligo, Ireland; h Mechanical Engineering Department, College of Engineering, King Khalid University, Abha 61421, Asir, Kingdom of Saudi Arabia; i Research Center for Advanced Materials Science (RCAMS), King Khalid University, Guraiger, Abha 61413, Asir, Kingdom of Saudi Arabia

**Keywords:** Rubidium cubic perovskite, Structural characteristics, Electronics characteristics, Optical characteristics, Elastic characteristics

## Abstract

Perovskite materials are considered the gateway of various physical applications to meet the production and consumption of energy and medical fields. Density Functional Theory (DFT) becomes the most important field in the modern era to investigate perovskite materials for various physical properties. DFT nowadays is used to explore the perovskite materials for a lot of applications like photocatalytic, optoelectronic, and photovoltaics. We discussed radium based cubic hydrides RbRaX_3_ (while X = F & Cl) perovskite material's electrical, optical, elastic, & physical characteristics with the help of DFT-based CASTEP code with PBE exchange-correlation efficient of GGA. The RbRaF_3_ & RbRaCl_3_ have three-dimensional nature by means of space group 221 (Pm3 m). According to electronic characteristics, the direct bandgap of RbRaF_3_ RbRaCl_3_ are 3.18eV and 2.209eV, respectively. Both compounds are brittle in nature via Poisson's ratio & Pugh's criteria. Thus, our novel RbRaX_3_ (X = F and Cl) compounds have excellent applications for solar cell and medical areas.

## 1 Introduction

Perovskites are well-known materials in the field of research, perovskite compounds are represented in general by ABX_3_ while A and B are cations having different sizes when X is a bound anion to both [1]. Organic photovoltaics, also known as OPVs, have a substantial amount of untapped potential to meet the ever-increasing energy needs of the future [2,3]. Extensive research on inorganic-organic perovskites has led to the identification of new resources that may be used in the creation of solar systems that are both effective and economical [4,5]. In 2009, many different perovskite-based photovoltaic cells (also known as perovskite solar cells, or PSCs) were reported to have fast-rising power conversion efficiencies (PCE) [[6], [7], [8]]. Perovskites through a piezoelectric effect for detectors, lead zirconium titanate, & high-temperature perovskite super-conductors such as beryllium copper oxide are a few examples [9,10]. When exposed to a magnetic field, some perovskite groups, primarily those based on manganese, exhibit extremely high resistance of magnet, which can expressively alter electrical resistance [11]. Furthermore, perovskites have been observed in a variety of other sectors, including lasers [12], light emitting diodes (LEDs), catalysts [13], and thermoelectric materials [14]. It has recently gained popularity due to capable of serving as a solar cell absorber and has gained large attention of researchers.

Fluoro-perovskites and halide perovskites are two categories of mixes with ABF_3_ and ABCl_3_ stoichiometry, respectively [15]. There is a large body of study on the crystals of halide perovskites and fluoro-perovskites [16]. In general, A is alkali, alkaline, or rare earth metals. Here B is considered as a transition, post-transition, & non-transition metal while X, which is oxides and halides, is used to represent an anion [17]. It has demonstrated that it can create a variety of chemically stable rising fluorides, the most prevalent of which are strong electro-positive alkali metals and alkaline earth metals. Due to their ferromagnetic [18], non-magnetic insulating material, piezoelectric, and photoluminescence properties, complex metal fluorides have grown a lot of interest [19]. But completely unleash organic photovoltaics' latent potential; it is still necessary to synthesize highly efficient photovoltaic materials, especially hole-transport materials (HTMs) and electron-transport materials (ETMs) [[20], [21], [22], [23]].

Moreover, numerous recent researches have demonstrated that the fluoro-perovskite crystals are guaranteed to have UV-Deep UV wave bands. KMgF_3_, NaSrF_3_, NaBaF_3_, and LiBaF_3_ can be used to create prisms, glasses, and windows that are fully transparent and optical with little loss. Halide perovskites are grabbing the interest of research teams due to their wide range of outstanding features, possible technical uses, & the chance to add almost each component throughout the periodic table [24,25]. Following these developments, research into the thickness-dependent optical, electronic, and vibrational properties of organic and hybrid perovskites has begun to gain momentum. Recent investigations have also demonstrated that these materials' optical, vibrational, and electronic properties may differ depending on their material thickness for solar cell applications. Due to its historic launch in solar cell (SC) applications, metal hydride perovskite have recently attracted enormous attention from the research community. These materials actually have intriguing optoelectronic characteristics such as a flexible band gap, a predominate point, and strong optical absorption defect for solar cell applications [26]. Therefore, in current study, we have attempted to improve the performance of electrical, optical, elastic, & physical characteristics with the help of DFT-based CASTEP code with PBE exchange-correlation efficient of GGA. Interestingly, we have found that the performances of the radium based cubic hydrides RbRaX_3_ (while X = F & Cl) perovskite materials for solar cell applications using DFT approach are significantly improved by Cl.

In this study, the structural, electrical, & optical characteristics of RbRaF_3_ and RbRaCl_3_ are studied. The whole energy estimation is achieved via DFT-designed GGA-PBE method, which is incorporated by CASTEP code. These compounds have great contribution for energy and medical applications.

## 2 Computational details

Both RbRaF_3_ and RbRacl_3_ are studied using cubic crystal structure of ternary fluoro perovskites. Both compounds belong to Pm3 m space group. In both compounds, atomic place of Rb atoms is (0.00, 0.00, 0.00) & the atomic position of F and cl atoms are (0.0, 0.5, 0.5). In RbRaF_3_ and RbRaCl_3_, the atomic positions of the Ra atom are (0.5, 0.5, 0.5). The following is the elemental configuration for the atoms in question: F: 2s2 2p^5^; Rb: 4s^2^ 4p^6^ 5s^1^ and Ra: 6s^2^ 6p^6^ 7s^2^ of RbRaF_3_ and Cl: 3s^2^ 3p^5^; Rb: 4s^2^ 4p^6^ 5s^1^ and Ra: 6s^2^ 6p^6^ 7s^2^ of RbRaCl_3_. To investigate the characteristics of our material, we employed the CASTEP program, which is developed on DFT [27]. The PBE functional are used to accomplish the calculations. We calculated the properties of our material using a unit cell. The characteristics of materials were calculated via geometry optimization. The total energy convergence per atom for RbRaF_3_ is 5 × 10^−6^ eV/atom and for RbRaCl_3_ is 1.0 × 10^−5^ eV/atom respectively. The atoms are subjected to extreme force of 0.010 eV/A and a max ionic displacement (5 × 10^−4^) for RbRaF_3_. In the case of RbRaCl_3,_ the max force and ionic displacement is set to 0.03 eV/A and 0.001 A respectively. In order to conduct an analysis of the material's band structure, k-integration was carried out on a mesh grid consisting of 8 × 8 × 8 k-points, and an energy cutoff of 230 eV was applied uniformly over the Brillouin zone. Our research outlines a method that is straightforward, economical, and kind to the natural world to prepare the effective perovskite materials discussed before. Aqueous lead precursors that do not include halides are subjected to a hydrothermal reaction during this method [20,23].

**Fig. 1:**
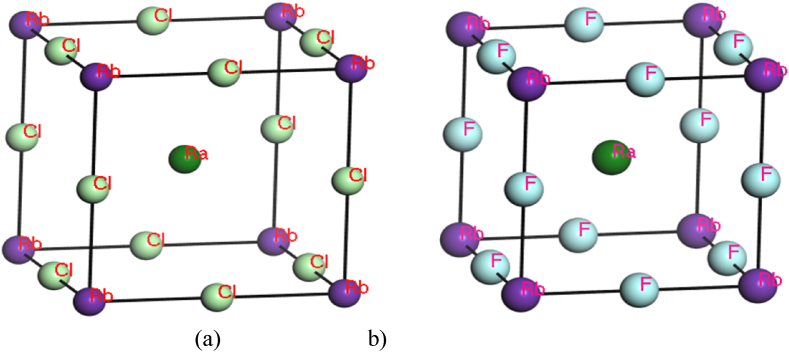
Crystal Structure of: (a) RbRaCl_3_ & (b) RbRaF_3_.

A limited experimental or theoretic data available for RbRaF_3_ & RbRaCl_3_ compounds. We have made a comparison with other Rb-based fluoro perovskites and halide perovskites. Table 1 shows the values of the lattice constant and band gap of RbRaCl_3_ and RbRaF_3_ in comparison with other Rb-based flour-perovskites materials. Fig. 1 (a) and Fig. 1(b) shows the crystal structure of RbRaCl_3_ and RbRaF_3_ compounds.

**Table 1:** Lattice Parameters, Volume, and Band gaps energy of RbRaF_3_ and RbRaCl_3_ compounds.

Compound used	Lattice Constant (A^0^)	V (A^0^)^3^	Band Gap (eV)
RbHgF_3_ [28]	4.60	97.33	3.2
RbMgF_3_ [29]	4.13	70.90	7.6
RbRaF_3_	3.55	44.73	3.18
RbRaCl_3_	6.45	268.71	2.209

## 3 Results and arguments

### 3.1 Structural properties

In order to discuss the structural properties, both substances' cell geometries are improved. Murnaghan state equation is utilized to derive balanced lattice limitations though keeping the overall energy of crystals minimal [30]. The value of the optimum lattice parameter is exposed to be over geometry optimization 3.55 A^0^ and 6.45 A^0^ for RbRaF_3_ and RbRaCl_3_ respectively as mentioned in Table 1. For both compounds, there is no theoretic or experimental information available in literature. Table 2 shows the values of calculated lattice constants and lists the values of these elastic constants. Three elastic constants, C11, C12, & C44, are used to explain the mechanical characteristics of cubic symmetry crystals.

**Table 2:** The computed elastic moduli, Poisson's ratio (v), Pugh's index ratio B/G, anisotropy factor A, and lattice constants Cij [GPa] at equilibrium volume.

Parameters	RbRaF_3_	RbRaCl_3_
C_11_	75.74	12.60
C_12_	−9.33	−1.08
C_44_	−0.87	−4.29
A	−61.09	−11.86
B	19.02	1.33
G	16.49	2.72
E	38.38	4.86
ѵ	0.16	−0.10
B/G	1.15	0.48

To calculate the bulk modulus, Equation (1) is used to find its values [31].

(1) B=(C_11_+2C_12_) / 3

For stability, Equation (2) is helpful to check the condition as follows: [32].

(2) C11 > 0; C44 > 0; (C11–C12)>0; (C11+2C12)>0; C12BCE11

In Table 2, effects of the anisotropy factor A is calculated from Equation (3), the young's modulus E is calculated from Equation (4), the possion ratio v is calculated from Equation (5), and the pugh's index ratio B/G is calculated from Equation (6), Equation (7) and Equation which are displayed as follows [33].

(3) A = (2C_4_) / (C_11_–C_12_)

(4) E = (9 B G) / (3B + G)

(5) v = (3B-2G) / 2(2B + G)

(6) G = (G_v_ + G_R_) / 2

(7) G_v_ = (C_11_–C_12_+3C_44_) / 5

(8) G_R_ = 5C_44_(C_11_–C_12_) / 4C_44_+3(C_11_–C_12_)

The B/G ratio is known as Pugh's ratio if its value is less than 1.750, the material is brittle, and for values more than 1.750, the material is supposed ductile [34]. The ratio of the poison can also be used to evaluate whether a material is harsh or ductile. Poisson's ratio v is another tool for determining if a substance is harsh or ductile; if this ratio rises above the threshold of 0.26, the substance is said to be ductile [35,36]. Table 2 shows the values for both Pugh's ratio and poison's ratio for RbRaF_3_ and RbRaCl_3_. Using these two indicators it can be concluded that both materials exhibit a brittle nature.

### 3.2 Electronic properties

**Fig. 2:**
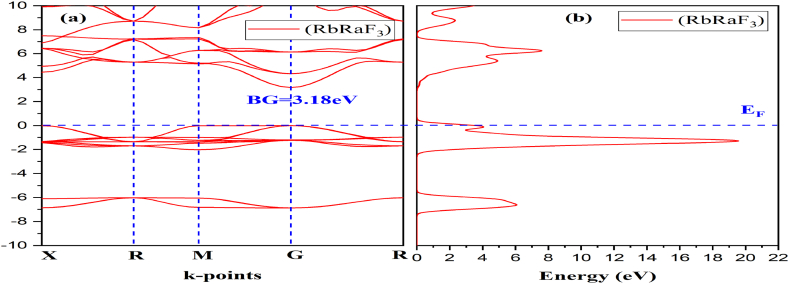
(a) Band structure (b) DOS of RbRaF_3_ compound.

When assessing the band structure of materials, the density of states, also known as DOS, is an essential component to take into consideration. Due to the existence of a bandgap at the Fermi levels, the DOS plot of RbRaF_3_ demonstrates unequivocally that the material has features typical of semiconductors [37]. The Rb-d orbital is responsible for the vast bulk of the contribution that is seen in the conduction band. On the other hand, the DOS plot of RbRaCl_3_ reveals semiconductor characteristics, with a bandgap located near the Fermi levels. The Rb-d orbital has the most significant impact on the conduction band, whereas the Rb-p and Ra-p orbitals have a marginal bearing on its characteristics. Fig. 2(a) shows the band structure while Fig. 2(b) depicts density of states of RbRaF_3_ compounds.

For the sake of our computations, the Fermi level is placed at the peak of the valence band and for the sake of clarity, only the states with the greatest contributions are observed.

**Fig. 3:**
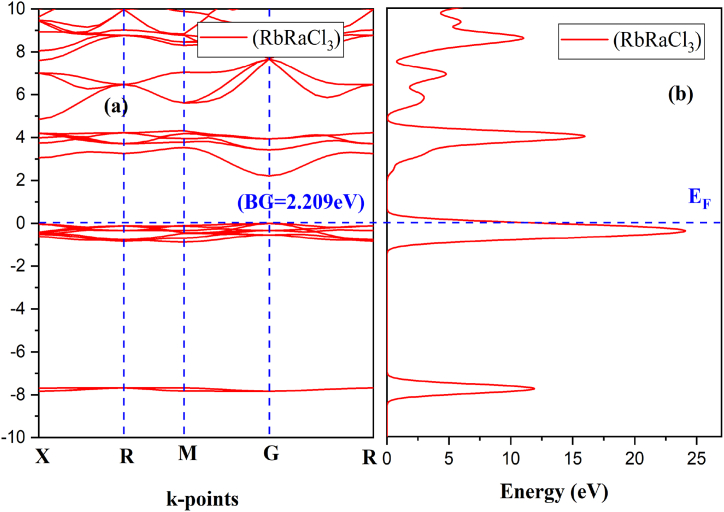
(a) Band structure (b) TDOS of RbRaCl_3_ compound.

**Fig. 4:**
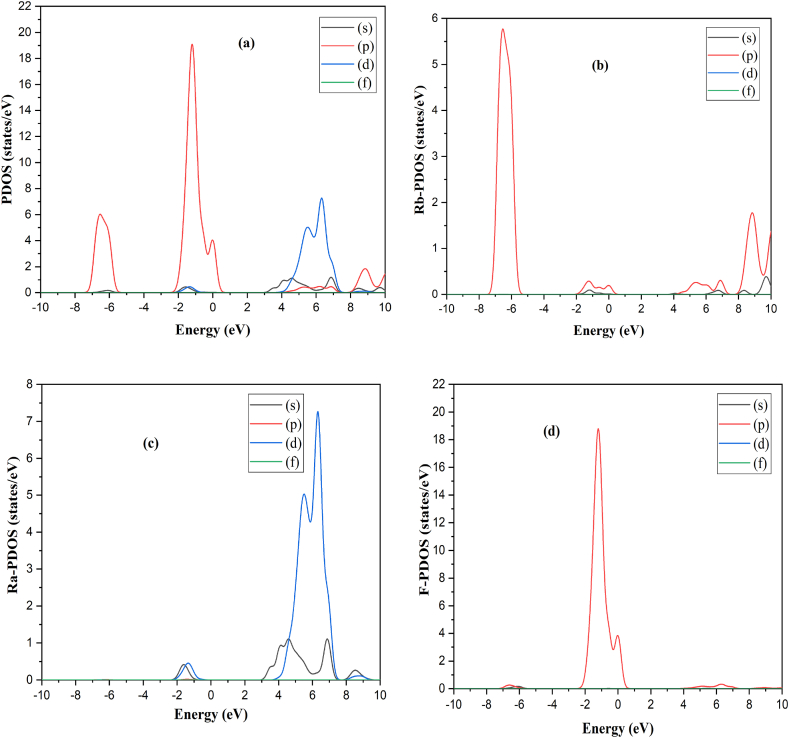
(a) PDOS of RbRaF_3_ compound. (b) Rb atoms- PDOS (c) Ra-PDOS (d) F- PDOS.

**Fig. 5:**
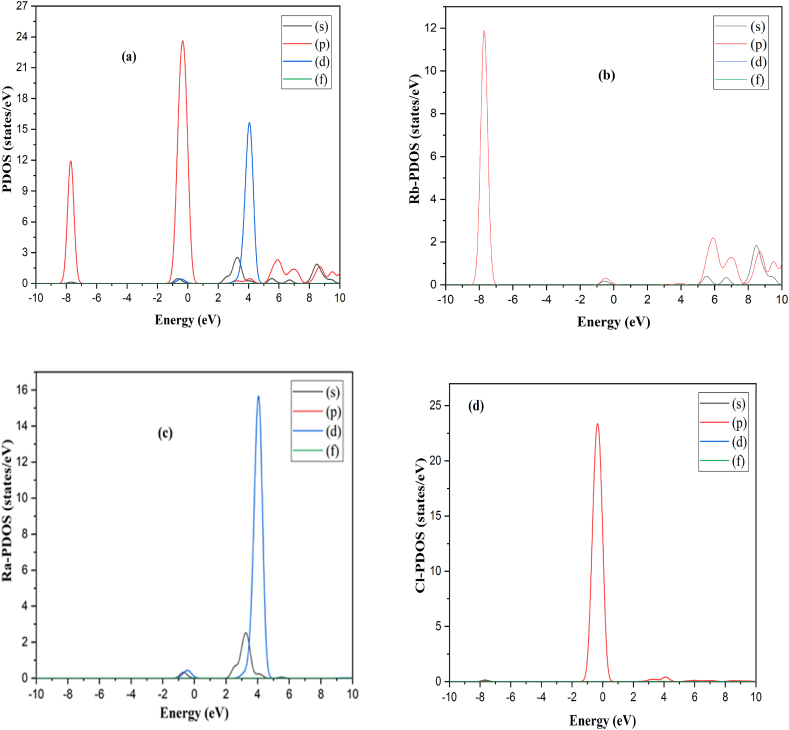
(a) PDOS of RbRaCl_3_ compound. (b) Rb- PDOS (c) Ra-PDOS (d) Cl – PDOS.

Moreover, it is possible to forecast a material's optical nature using its electrical properties [38,39]. Thus, we performed the BG and DOS calculations of RbRaF_3_ and RbRaCl_3_ to comprehend their optical and electrical characteristics. Fig. 3(a)–(b) show band structures and TDOS of RbRaF_3_ and RbRaCl_3_ respectively. The E_F_ was fixed to 0.0 eV which coincides with the top of valence band. The calculated bandgap of RbRaF_3_ and RbRaCl_3_ is 3.18 eV and 2.209 eV correspondingly. At the G point in both valence band maximum and conduction band minimum are observed. RbRaF_3_ & RbRaCl_3_ are showing that both compounds have a direct band gap [40]. As a result, these materials may be suitable for optoelectronics, photovoltaic, and photo-thermal applications. Fig. 4 (a) shows PDOS of RbRaF_3_ compounds. The p-states of the fluorine and Rb atoms, respectively, contribute significantly to the formation of the valence band as observed in Fig. 4(b). The conduction band is formed by the main role of Ra-d states & the minor involvement of Ra- s-states & Rb-p states as observed in Fig. 4(c). Moreover, Fig. 4(d) shows F- PDOS formation. Fig. 5 (a) shows PDOS of RbRaCl_3_ compound. In RbRaCl_3_ below the fermi level, the major contribution is due to Cl-p states and Rb-states as observed in Fig. 5(b)–(d). Above fermi level, conduction band is mostly formed due to the major contribution of Ra-d states and minor contribution of Rb-p states and Ra –states as seen in Fig. 5(c).

### 3.3 Optical properties

**Fig. 6:**
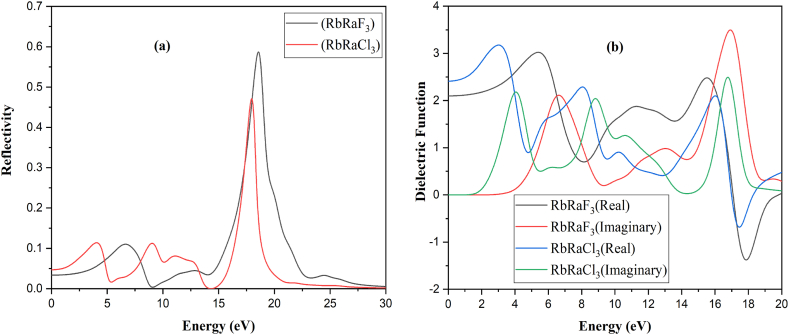
(a) Reflectivity and (b) Dielectric Function of RbRaF_3_ and RbRaCl_3_ compounds.

The optical properties of compounds including their reflectivity, energy loss function, relative permittivity, and index of refraction, can be utilized to define their electronic structure. These qualities are highly useful in determining the material's suitability and feasibility in nano-electronics and optoelectronics. Fig. 6(a) and (b) show the optical characteristics of RbRaF_3_ and RbRaCl_3_ compounds. Because all of the optical properties are interrelated, dielectric function *ε* (ω) is used, which is calculated by Equation (9) [41]:

(9) *ε*(ω) = *ε*_1_ (ω) + iε_2_ (ω)

In the above equation, *ε*_1_ (ω) denotes the real part while *ε*_2_ (ω) signifies the imaginary portion of the dielectric function.

Further, optical properties such as n (ω) is calculated by Equation (10), L (ω) is calculated by Equation (11), I (ω) is calculated by Equation (12), and R (ω) is calculated by Equation (13) as follows: [42].

(10) n(ω) = [ *ε*_1_(ω)/2 + { *ε*^2^_1_(ω) + *ε*^2^_1_ (ω) }^1/2^ /2 ]^1/2^

(11) L(ω) = − I m (*ε*(ω)−^1^) = *ε*_2_(ω) / *ε*_1_(ω) ^2^ + *ε*_2_(ω) ^2^

(12) I(ω) = 2^1/2^ ω [{ *ε*^2^_1_ (ω) + *ε*^2^_1_(ω) }^1/2^ − *ε*1(ω) ]^1/2^

(13) R(ω) = (n + ik − 1) / (n + ik + 1)

The main reflectivity peak for RbRaF_3_ seems at 0.57 at 18.52eV while that for RbRaCl_3_ appears at 0.46 at 18.0eV. The rate of reflectivity for RbRaF_3_ is 0.03 & for RbRaCl_3_ is 0.04 at 0eV. The reflectivity is zero at 9.09 eV and 14.52 eV of RbRaF_3_ and RbRaCl_3_.

**Fig. 7:**
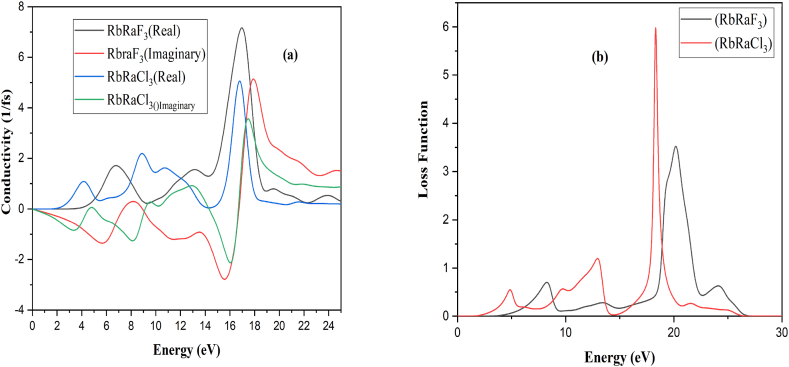
(a) Conductivity and (b) Absorption of RbRaF_3_ and RbRaCl_3_ compounds.

The conductivity's primary peak (Real) is 7.10 at 17.0 eV and 5.00 at 16.72 eV of RbRaF_3_ and RbRaCl_3_ and the imaginary part of the conductivity is 5.14 at 18.0eV and 3.49 at 17.48eV of RbRaF_3_ and RbRaCl_3_. Fig. 7(a) shows that initially the conductivity of the compounds will be increased gradually and after reaching at maximum peak the conductivity start to decrease.

The main absorption peak for RbRaF_3_ is 17.55 eV while for RbRaCl_3_ it is 17.15 eV. Fig. 7(b) depicts that absorption has no value when 2.24 eV and 4.00 eV for RbRaF_3_ and RbRaCl_3_. The absorption of the compounds increases and after the maximum peak, it starts to decrease.

**Fig. 8:**
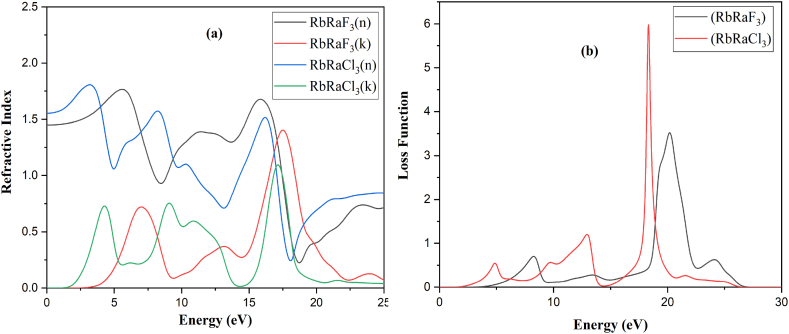
(a) Refractive index and (b) loss function of RbRaF_3_ and RbRaCl_3_.

The primary maximum of the refractive index (n) for RbRaF_3_ is 1.76 at 5.58 eV whereas for RbRaCl_3_ is 1.80 at 3.11 eV. Fig. 8(a) demonstrates that the refractive index (k) for the primary peak for RbRaF_3_ is 1.39 at 17.48 eV while that for RbRaCl_3_ is 1.06 at 17.10 eV. At 0.0 eV, the value of the refractive index (n) for RbRaF_3_ is 1.43 whereas for RbRaCl_3_ is 01.53. Refractive index (k) starts at zero at 1.56eV and 3.29 of RbRaCl_3_ and RbRaF_3_. Real part of refractive index initially increases and then starts to decrease. The primary dielectric function peak (real) for RbRaF_3_ appears a 2.14 and 2.07 at 0 eV of RbRaF_3_ and RbRaCl_3_ and the imaginary part appears at 0.0 at 1.53eV and 3.28eV of RbRaF_3_ and RbRaCl_3_ compounds. The major ultimate of dielectric function (for imaginary) for RbRaF_3_ give the impression at 3.02 at 5.33eV while that for RbRaCl_3_, it seems at eV.

The rate of the loss function of main peak is 3.49 at 20.17 eV and 5.90 at 18.29 eV for RbRaF_3_ and RbRaCl_3_ as observed in Fig. 8(b). The loss function starts to rise at 4.42 eV and 2.52 eV of RbRaF_3_ and RbRaCl_3_ and increases gradually and decreases to zero.

## 4 Overview

In order to discuss the overview of the perovskites materials, following steps are necessary.

1. Degradation issue of CH_6_I_3_NPb perovskite needs to be studied.
2. The film quality and thickness are core problems in perovskite solar cells.
3. Perovskite materials will break down quickly due to exposure of heat, moisture and snow.
4. The material is toxic in nature. The above mentioned points are the limitations of perovskite solar cell.

In addition, two basic strategies have been discovered as ways to improve the perovskite cells' capacity to maintain their stability. The first strategy involves the addition of composite perovskite components in order to boost the perovskite's natural stability. The second strategy involves finding appropriate additive compounds that may effectively limit the perovskite materials' breakdown. This strategy has the potential to be more successful than the first.

## 5 Conclusion

In modern period, perovskite materials play an excellent role for many applications that can be used to investigate photocatalytic, optoelectronic, energy and medical applications. In this study, we investigated Rubidium-based hydride perovskite materials via first-principles calculations. CASTEP code is used for the investigation of RbRaF_3_ and RbRaCl_3_ compounds, with cutoff energy 230 eV and 8 × 8 × 8 mesh k-points. The structural, mechanical, optical, and electrical properties of RbRaF_3_ and RbRaCl_3_ compounds are reported. We observed that both compounds exhibit brittle nature according to the Possion ratio and Pugh ratio calculations. It is realized that both materials have direct band gaps and the band gaps' values are found to be 3.18 and 2.209 for RbRaF_3_ and RbRaCl_3,_ respectively. The main absorption peak for RbRaF_3_ is 17.55 eV while for RbRaCl_3_ it is 17.15 eV. In connection to structural-electronic purpose, all-optical properties including energy loss function, absorption, reflection, & refractive index are studied. This work represents significant progress towards energy and medical applications.

## Author contribution statement

Muhammad Khuram Shahzad: Conceived and designed the experiments.

Muhammad Umair Farooq, Rashid Ali Laghari: Analyzed and interpreted the data.

Muhammad Aslam Khan, Muhammad Bilal Tahir, Waqar Azeem, Vineet Tirth: Contributed reagents, materials, analysis tools or data.

Muhammad Mahmood Ali: Performed the experiments; Wrote the paper.

## Data availability statement

Data will be made available on request.

## Funding information

Deanship of Scientific Research at King Khalid University Abha 61,421, Asir, Kingdom of Saudi Arabia Large Groups Project under grant number RGP.2/499/44.

## Declaration of competing interest

The authors declare that they have no known competing financial interests or personal relationships that could have appeared to influence the work reported in this paper.
